# Prevalence and antimicrobial susceptibility profile of *Salmonella* isolated from vegetable farms fertilized with animal manure in Addis Ababa Ethiopia

**DOI:** 10.1038/s41598-024-70173-4

**Published:** 2024-08-19

**Authors:** Woinshet Hailu, Haile Alemayehu, Deneke Wolde, Lulit Hailu, Girmay Medhin, Gireesh Rajashekara, Wondwossen A. Gebreyes, Tadesse Eguale

**Affiliations:** 1 Aklilu Lemma Institute of Pathobiology, Addis Ababa University, Addis Ababa, Ethiopia; 2 College of Health Sciences, Addis Ababa University, Addis Ababa, Ethiopia; 3 Department of Medical Laboratory Science, College of Medicine and Health Sciences, Wachemo University, P.O. Box 667, Hossana, Ethiopia; 4 Ethiopian Public Health Institute, Addis Ababa, Ethiopia; 5 Global One Health Initiative (GOHi), Ohio State University, Columbus, OH USA; 6 Center for Food Animal Health, Department of Animal Sciences, College of Food, Agricultural, and Environmental Sciences, The Ohio State University, Wooster, OH USA; 7 Department of Preventive Veterinary Medicine, College of Veterinary Medicine, The Ohio State University, Columbus, OH USA; 8 Ohio State University Global One Health LLC, Addis Ababa, Ethiopia

**Keywords:** Animal manure, Antimicrobial resistance, Foodborne pathogens, *Salmonella*, Microbiology, Environmental sciences

## Abstract

The resistance of foodborne pathogens to antimicrobial agents is a potential danger to human health. Hence, establishing the status of good agricultural practices (GAPs) and the antimicrobial susceptibility of major foodborne pathogens has a significant programmatic implication in planning interventions. The objective of this study was to assess the gap in attaining GAP and estimate the prevalence and antimicrobial susceptibility profile of *Salmonella* in vegetable farms fertilized with animal manure in Addis Ababa, Ethiopia. A total of 81 vegetable farms from four sub-cities in Addis Ababa were visited, and 1119 samples were collected: soil (n = 271), manure (n = 375), vegetables (n = 398), and dairy cattle feces (n = 75). Additional data were collected using a structured questionnaire. Isolation of *Salmonella* was done using standard microbiology techniques and antimicrobial susceptibility testing was conducted using disk diffusion assays. Carriage for antimicrobial resistance genes was tested using polymerase chain reaction (PCR). Among the 81 vegetable farms visited, 24.7% used animal manure without any treatment, 27.2% used properly stored animal manure and 80.2% were easily accessible to animals. The prevalence of *Salmonella* was 2.3% at the sample level, 17.3% at the vegetable farm level, and 2.5% in vegetables. The highest rate of resistance was recorded for streptomycin, 80.7% (21 of 26), followed by kanamycin, 65.4% (17 of 26), and gentamicin, 61.5% (16 of 26). Multidrug resistance was detected in 61.5% of the *Salmonella* isolates. Vegetable farms have a gap in attaining GAPs, which could contribute to increased contamination and the transfer of antimicrobial resistance to the vegetables. The application of GAPs, including proper preparation of compost and the appropriate use of antimicrobials in veterinary practices, are recommended to reduce the emergence and spread of antimicrobial resistance.

## Introduction

Foodborne infections are major public health problems worldwide^1^. For a healthy lifestyle, eating vegetables is encouraged to prevent different chronic diseases^2^. However, the number of foodborne outbreaks caused by contaminated vegetables is increasing from time to time^3^. Animal manure, which is widely used as fertilizer in different parts of the world to increase agricultural production^4^, can be contaminated with foodborne bacteria such as *Salmonella*, *Escherichia coli*, and *Campylobacter*, increasing the risk of infection by these pathogens^5^. *Salmonella* is the leading cause of produce-related outbreaks worldwide^6^.

The application of animal manure as fertilizer has been reported to contaminate soil with antimicrobial agents discharged from the feces or urine of animals^7^. In addition to the presence of foodborne pathogens in the food chain, antimicrobial-resistant bacteria carrying different genetic resistance markers enter the human food chain from food animals subjected to regular antibiotic treatments^8^. This is a major health concern because many of the antimicrobial agents used in animal production and human therapeutics are analogues^9^. These analogue antibiotics may select for resistant phenotypes of the pathogen that could cause more antibiotic-resistant bacterial illnesses in humans^10^.

The application of animal manure to agricultural soil during the production of food crops to improve soil fertility is a routine practice in many countries around the world, including Ethiopia^11,12^. In developing countries, animal manure is a major nutrient source for smallholder farmers who cannot afford mineral fertilizers^4^. In urban and peri-urban Addis Ababa, there are small-scale farms in close proximity to human residential areas, and in some cases, animal manure is used as fertilizer for the growth of different vegetables^13^.

Multiple studies have shown the occurrence of *Salmonella* and the increase in antimicrobial resistance among *Salmonella* isolates in Ethiopia. For instance, the prevalence of *Salmonella* in poultry farms was 14.6%^14^, and that in dairy farms was 8.3%^15^. The prevalence of *Salmonella* in diarrheic patients ranges from 1.1 to 6.2%^16,17^. In addition, several studies in Ethiopia have reported a high rate of antimicrobial resistance in *Salmonella* isolates from different sources^18,19^. However, there is a lack of data showing the impact of using animal manure as fertilizer on the dissimilation of *Salmonella*.

Here, we investigated the prevalence and antimicrobial resistance profile of *Salmonella* from animal manure amended vegetable farms in highly populated urban and peri-urban Addis Ababa, where vegetable farms are in close proximity to animals and humans. The good agricultural practices (GAPs) of these vegetable farms were also assessed.

## Materials and methods

### Study area and study design

This study was conducted in Addis Ababa, Ethiopia. Addis Ababa is the capital city of Ethiopia, located at 9° 1′ 48″ N, 38° 44′ 24″ E. The average altitude of Addis Ababa is 2400 m above sea level, with the highest elevation at Entoto Hill to the north reaching 3200 m. It has a subtropical highland climate, with average annual temperature of 16.3 °C and 1089 mm annual rainfall^20^. A cross-sectional study was conducted from February 2022 to March 2023.

### Study farms

Four of the 11 sub-cities in Addis Ababa: Akaki-Kality, Nifas-Silk Lafto, Arada, and Gulelle sub-cities were randomly selected of which vegetable farms available in these sub-cities that reported the use of animal manure as fertilizer were recruited. Based on the availability of farms in each sub-city, a total of 81 vegetable farms were visited: 41 from the Akaki Kality sub-city, 23 from Nefas-Silk Lafto, 14 from the Gulele sub-city, and 3 from the Arada sub-city as shown in our previous study^21^.

### Data and sample collection

A farm review consisting of onsite observation and face-to-face interviews with vegetable farmers was conducted in the visited 81 vegetable farms. The farm review was based on the USA Department of Agriculture (USDA) checklist for Good Agricultural Practices (GAPs)^22^. The focus was given to the source of water, animal activity, and handling of compost manure as part of the WHO requirement to grow safer vegetables^1^.

Manure, soil, and vegetable sample collection was performed as previously described^23,24^. Briefly, from each vegetable farm, 15 surface subsamples (0–20 cm) (3 plots of 1 m^2^ each, per farm, 6 m apart from each other, and 5 subsamples from each plot, including the four corners and the middle of the plot) were collected and made into a single composite soil. Six manure samples were collected from each farm and pooled to obtain one composite manure sample. The same sampling technique was followed, except that only six subsamples (for each vegetable type) were used in the case of vegetables. Moreover, fresh fecal droppings from dairy cattle living around vegetable farms were also collected.

A total of 1119 samples were collected: 521 samples from Akaki Kality, 268 from Nefas Selk Lafto, 258 from Gulele, and 72 from Arada. Based on sample type, 398 were vegetables, 375 were manure, 271 were soil, and 75 were dairy cattle feces. The number and type of samples collected in each sub-city are shown in Table 1.

**Table 1:** Number of vegetable farms visited in each sub city and type of the collected sample.

Sub city	No. of farms	No. of samples collected	Sample type						
			Manure	Soil	Feces	Vegetables			
						Kale	Cabbage	Lettuce	Swiss chard
Gulele	14	258	87	79	20	33	1	24	14
Arada	3	72	19	22	-	7	2	18	4
Akaki Kality	41	521	174	115	39	52	35	74	32
Nefas Selk Lafto	23	268	95	55	16	28	15	37	22
Total	81	1119	375	271	75	120	53	153	72

Then samples were transported to the Microbiology Laboratory of the Aklilu Lemma Institute of Pathobiology, Addis Ababa University, in an icebox containing an ice pack and processed within 4–6 h of collection.

### Isolation and identification of *Salmonella*

*Salmonella* was detected using the guidelines of the International Organization for Standardization (ISO) 6579^25^. Ten grams of each sample was suspended in 90 mL of Buffered Peptone Water (BPW) and mixed by shaking to form a slurry. The slurry was incubated at 37 °C for 24 h, and 1 mL of the slurry was enriched in 10 mL tetrathionate broth (Oxoid Ltd., Cambridge, UK) and incubated at 37 °C for 24 h. One hundred µL of the grown culture was then transferred to 9.9 mL of Rappaport–Vassiliadis broth (RV; Oxoid Ltd., Cambridge, UK) and incubated at 42 °C for 24 h. A loopful of bacteria grown in RV were plated on XLT-4 plates and incubated at 37 °C for 24 h^26^. Identification of presumptive *Salmonella* colonies and biochemical tests were performed as described previously^27^.

Isolates showing specific biochemical characteristics of *Salmonella* were further confirmed using *Salmonella* genus-specific PCR as previously described^28^. PCR was based on the amplification of a 496-base pair (bp) segment of histidine transport operon gene, which is highly conserved among species of *Salmonella*. The forward and reverse primer sequences, from 5′ to 3′ were ACTGGCGTTATCCCTTTCTCTGGTG; and ATGTTGTCCTGCCCCTGGTAAGAGA. The reference strain of *Salmonella enterica* serovar Typhimurium ATCC 13311 was used as a positive control. PCR confirmed *Salmonella* isolates were preserved at − 80 °C in 20% glycerol until further analysis.

### Antimicrobial susceptibility testing

Antimicrobial susceptibility testing of the *Salmonella* isolates was performed according to the Clinical Laboratory Standards Institute guideline^29^ using the Kirby-Bauer disk diffusion method on Muller-Hinton agar plates (Oxoid, CM0337 Basingstoke). The antimicrobial agents used in the present study were ampicillin (10 μg), nalidixic acid (30 µg), sulfamethoxazole + trimethoprim (1.25/23.75 μg), sulfisoxazole(1000 μg), chloramphenicol (30 μg), ceftriaxone (30 μg), amoxicillin + clavulanic acid (20/10 µg), streptomycin (10 μg), kanamycin (30 μg), ciprofloxacin (5 μg), tetracycline (30 μg), gentamicin (10 μg), amikacin (30 μg) and azithromycin (15 μg). The antimicrobial disks used in this study were all from Sensi-Discs (Becton, Dickinson, Company, Loveton, USA).

*Salmonella* isolates were considered multidrug-resistant when they were resistant to three or more antimicrobial agents belonging to different classes^30^. *Escherichia coli* ATCC 25922 was used as a quality control strain when conducting antimicrobial susceptibility tests.

*Salmonella* isolates that were phenotypically resistant to tetracycline, ampicillin, gentamycin, streptomycin and sulfonamide were screened for: tetracycline resistant genes (*tet(A), tet(B),* and *tet(C)),* aminoglycoside resistance genes (aminoglycoside acetyltransferase genes (*aac (3)-IV)*, and adenyl transferase gene (*aadA)*, sulfonamide resistant genes (*sulI* and *sulII)*, and beta-lactamase gene (*bla *_*TEM*_), using conventional PCR. The PCR conditions and primer sequences are described previously^21^. The PCR product was observed using agarose gel electrophoresis.

### Statistical analysis

Descriptive statistical methods were used to summarize the characteristics of the farms. The chi-square test was used to assess the associations of different factors with *Salmonella* positivity. A p-value less than 0.05 was considered to indicate a statistically significant association. SPSS version 26 was used to perform the descriptive analysis and the association between the antimicrobial resistance pattern, their genetic determinants, and the source of the isolates was assessed as described previously^21^.

### Ethical consideration

The Institutional Review Board of Aklilu Lemma Institute of Pathobiology reviewed the protocol for its ethical and methodological standards and approved the conduct of the study (Ref. No. ALIPB IRB/75/2014/22 on April 30, 2022). The plant collection and use was in accordance with all the relevant guidelines.

## Results

### Vegetable farm description and farm practices

Most of the vegetable farms, 95% (77 of 81) used dairy manure, while only 5% (4 of 81) used poultry litter to fertilize their farms. The majority (88.8%; 71/88) applied both organic and inorganic fertilizers, and the remaining 11.1% (9 of 81) used only organic fertilizer. Of the 81 vegetable farms visited, 75.3% (61 of 81) used compost, and the remaining 24.7% (20 of 81) of the farmers applied animal manure without any treatment. Only 27.2% (22 of 81) of the vegetable farms stored manure properly to prevent contamination.

Most of the vegetable farms (80.2%; 65/81) were not fenced and were easily accessible to domestic and wild animals. A total of 92.6% (75 of 81) of the vegetable farms reported that they use river water and 7.4% (6 of 81) of the farms use both rain water and tap water to irrigate their farms.

There are no steps taken to protect irrigation water from contamination or to restrict livestock access to the source or delivery of irrigation water. There was no monitoring system on the farms for the presence of animals entering the vegetable farms. Most of the vegetable farms use composted manure to fertilize their farms 75.3% (61 of 81), whereas 24.7% (20 of 81) farms use fresh, untreated manure. Only 27.2% (22 of 81) of the vegetable farms stored manure properly to prevent contamination (Table 2).

**Table 2:** Results of farm review checklist for good agricultural practices (GAPs). Number of farms studied (n = 81).

No	Farm aspect of good agricultural practice activity question	Number of farms in each category (%)		
		Yes	No	NA
Water usage				
1	If the farm use irrigation, are steps taken to protect irrigation water from potential direct and nonpoint source contamination	0	76 (93.8)	5 (6.2)
Sewage treatment				
2	There is no municipal/commercial sewage treatment facility or waste material landfill adjacent to the farm	55 (67.9)	26 (32.1)	0
Animal/wildlife/livestock				
3	Vegetable farm areas are not located near or adjacent to dairy, livestock, or poultry production facilities unless adequate barriers exist	49 (60.5)	32 (39.5)	0
4	Manure lagoons located near to the vegetable farm areas are maintained to prevent leaking/overflowing, or measures have been taken to stop runoff from contaminating the vegetable farm areas	16 (19.8)	27 (33.3)	38 (46.9)
5	Manure stored near or adjacent to vegetable farm areas is contained to prevent contamination of crops	22 (27.2)	27 (33.3)	32 (39.5)
6	Measures are taken to restrict access of livestock to the source or delivery system of crop irrigation water	0	0	81(100)
7	Vegetable farm areas are monitored for the presence or signs of wild or domestic animals entering the land	0	0	81(100)
8	Measures are taken to reduce the opportunity for wild and/or domestic animals from entering vegetable farm areas	16 (19.8)	65 (80.2)	0
Manure option A: raw manure				
9	When raw manure is applied, it is incorporated at least 2 weeks prior to planting or a minimum of 120 days prior to harvest	20 (24.7)	61 (75.3)	0
10	Raw manure is not used on commodities that are harvested within 120 days of planting	20 (24.7)	61 (75.3)	0
11	If both raw and treated manure are used, the treated manure is properly treated, composted or exposed to reduce the expected levels of pathogens	12 (14.8)	69 (85.2)	0
12	Manure is properly stored prior to use	10 (12.3)	71 (87.7)	0
Manure option B: composted manure				
13	Only composted manure is used as a soil amendment	61 (75.3)	20 (24.7)	0
14	Composted manure is properly treated, composted, or exposed to environmental conditions that would lower the expected level of pathogens	26 (32.1)	55 ( 67.9)	0
15	Composted manure is properly stored and are protected to minimize recontamination	12 (14.8)	69 (85.2)	0

*NA* not applicable.

### Prevalence and distribution of *Salmonella*

The farm level prevalence of *Salmonella* in vegetable farms was 17.3% (14 of 81). In terms of sub-city stratification, 17.0% (7 of 41) of the vegetable farms in Akaki Kality, 21.7% (5 of 23) of the vegetable farms in Nefas Selk Lafto, and 14.3% (2 of 14) of the vegetable farms in the Gulele sub-city were positive for *Salmonella*. The presence of *Salmonella* in vegetable farms was not significantly associated with any of the studied factors or sub-city classifications (p > 0.05).

The sample-level prevalence of *Salmonella* was 2.3% (26/1119). The 26 *Salmonella* isolates were obtained from samples collected in the Gulele sub-city (4/258; 1.6%), Akaki Kality sub-city (13/521; 2.5%), and Nefas Selk Lafto sub-city (9/268; 3.3%), and no *Salmonella* was isolated from the Arada sub-city. The prevalence of *Salmonella* on vegetable farms is summarized in Table 3.

**Table 3:** Prevalence of *Salmonella* in vegetable farms in Addis Ababa, Ethiopia.

Sub city	No of vegetable farms	No of samples	No (%) of positive samples	No (%) of positive farms
Gulele	14	258	4 (1.6)	2 (14.3)
Arada	3	72	–	–
Akaki kality	41	521	13 (2.5)	7 (17.0)
Nefas Selk Lafto	23	268	9 (3.3)	5 (21.7)
Total	81	1119	26 (2.3)	14 (17.3)

**Figure 1:**
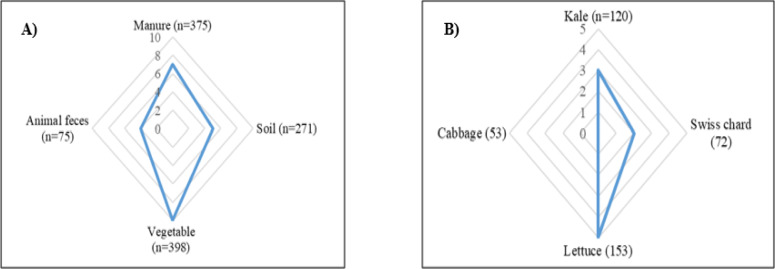
Radar map of (**A**) number of *Salmonella* positive samples: manure, soil, feces, and vegetables; and (**B**) number of *Salmonella* positive vegetables: kale, cabbage, swiss chard, and lettuce.

The highest prevalence (5.3%; 4/75) was detected in dairy cattle feces, followed by vegetables (2.5%; 10/398). A similar prevalence (1.8%) was detected in manure (7/375) and soil (5/271) samples. From the total of 398 vegetable samples, the highest prevalence was recorded in lettuce at 3.2% (5 of 153). However, the prevalence of *Salmonella* in kale was 2.5% (3 of 120) and, that in Swiss chard was 2.7% (2 of 72). *Salmonella* was not isolated from cabbage. The prevalence of *Salmonella* in different sample types and vegetables is shown in Fig. 1.

### Antimicrobial susceptibility profile of *Salmonella*

**Figure 2:**
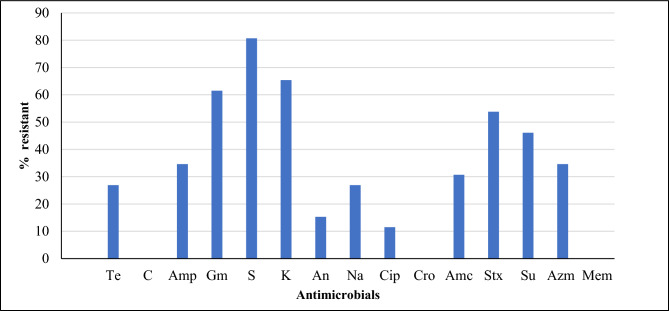
Antimicrobial resistance profile of *Salmonella* from vegetable farms in Addis Ababa, Ethiopia. *Te* tetracycline, *C* chloramphenicol, *Amp* ampicillin, *Gm* gentamicin, *S* streptomycin, *K* kanamycin, *An* amikacin, *Na* nalidixic acid, *Cip* ciprofloxacin, *Cro* ceftriaxone, *Amc* amoxicillin + clavulanic-acid, *Stx* sulfamethoxazole + trimethoprim, *Su* sulfisoxazole, *Azm* azithromycin, *Mem* meropenum.

Regardless of the source, all the *Salmonella* isolates were susceptible to chloramphenicol, ceftriaxone and meropenem. The highest resistance rate was recorded for streptomycin (80.7%, 21 of 26), followed by kanamycin (65.4%, 17 of 26), and gentamicin (61.5%, 16 of 26). The resistance to amoxicillin + clavulanic, amikacin, and ciprofloxacin was 30.7% (8 of 26), 15.3% (4 of 26), and 11.5% (3 of 26), respectively. Resistance to sulfamethoxazole + trimethoprim was 53.8% (14 of 26), and that of sulfisoxazole was 46.1% (12 of 26) (Fig. 2). A similar resistance rate of 26.9% (7 of 26) was recorded for tetracycline and nalidixic acid and 34.6% (9 of 26) for ampicillin and azithromycin. Multidrug resistance was observed in 61.5% (16 of 26) of the *Salmonella* isolates.

### Antimicrobial resistance genotypic profile of the *Salmonella* isolates

Resistance genes encoding for aminoglycoside resistance *aadA* were detected in 76.2% (16 of 21) of tested isolates, followed by *bla*_*TEM*_ detected in 66.6% (6 of 9), and *aac(3)-IV* was detected in 62.5% (10 of 16) of isolates. On the other hand, *tet (A)* and *tet (B)* were detected in 28.6% (2 of 7), and 57.1% (4 of 7), respectively, and *tet (C)* gene was detected in none of the isolates. Moreover, *sulI* and*, sulII* were detected in 28.6% (4 of 14) and 50% (7 of 14), of the strains, respectively. The detailed distribution of AMR genes among *Salmonella* isolates from different sample sources is shown in Table 4.

**Table 4:** Prevalence of selected antimicrobial resistance genes in *E. coli* isolates obtained from manure, soil, feces and vegetables in Addis Ababa, Ethiopia.

Encode resistance to	Source of *Salmonella* isolates	No. of resistant isolates	Tested resistant genes	No. of positive (%)
Tetracycline	Manure	4	*tet (A)*	1 (25)
			*tet (B)*	3 (75)
			*tet (C)*	0
	Soil	1	*tet (A)*	0
			*tet (B)*	1 (100)
			*tet (C)*	0
	Vegetables	2	*tet (A)*	1 (50)
			*tet (B)*	0
			*tet (C)*	0
	Feces	0	*tet (A)*	0
			*tet (B)*	0
			*tet (C)*	0
Ampicillin	Manure	3	*bla*_*TEM*_	3 (100)
	Soil	1	*bla*_*TEM*_	1(100)
	Vegetables	2	*bla*_*TEM*_	1 (50)
	Feces	3	*bla*_*TEM*_	1 (33.3)
Gentamicin	Manure	6	*aac(3)-IV*	3 (50)
	Soil	2	*aac(3)-IV*	1 (50)
	Vegetables	7	*aac(3)-IV*	5 ()
	Feces	1	*aac(3)-IV*	1(100)
Streptomycin	Manure	7	*aadA*	4 (57.1)
	Soil	4	*aadA*	4(100)
	Vegetables	6	*aadA*	4 (66.6)
	Feces	4	*aadA*	4 (100)
Sulfonamide	Manure	4	*sulI*	2 (50)
			*sulII*	1 (25)
	Soil	5	*sulI*	0
			*sulII*	4 (80)
	Vegetables	3	*sulI*	1 (33.3)
			*sulII*	2 (66.6)
	Feces	2	*sulI*	1 (50)
			*sulII*	0

### Correlation between phenotypic and genotypic resistance profiles of *Salmonella* isolates

Hierarchal clustering showed that eleven *Salmonella* isolates that were susceptible to tetracycline were clustered together (Cluster A). Moreover, six isolates in this cluster were streptomycin resistant and contained the *aadA* gene. Another nine *Salmonella* isolates susceptible to nalidixic acid and ciprofloxacin and one tetracycline resistant isolate were clustered together (Cluster B). In Cluster B, there were six *Salmonella* isolates that were resistant to gentamycin and contained *aac(3)-IV.* Six isolates in Cluster B were from vegetable samples, one from a fecal sample and two from a manure sample. Similarly, six *Salmonella* isolates were clustered together, all of which were resistant to streptomycin and kanamycin (Cluster C).

**Figure 3:**
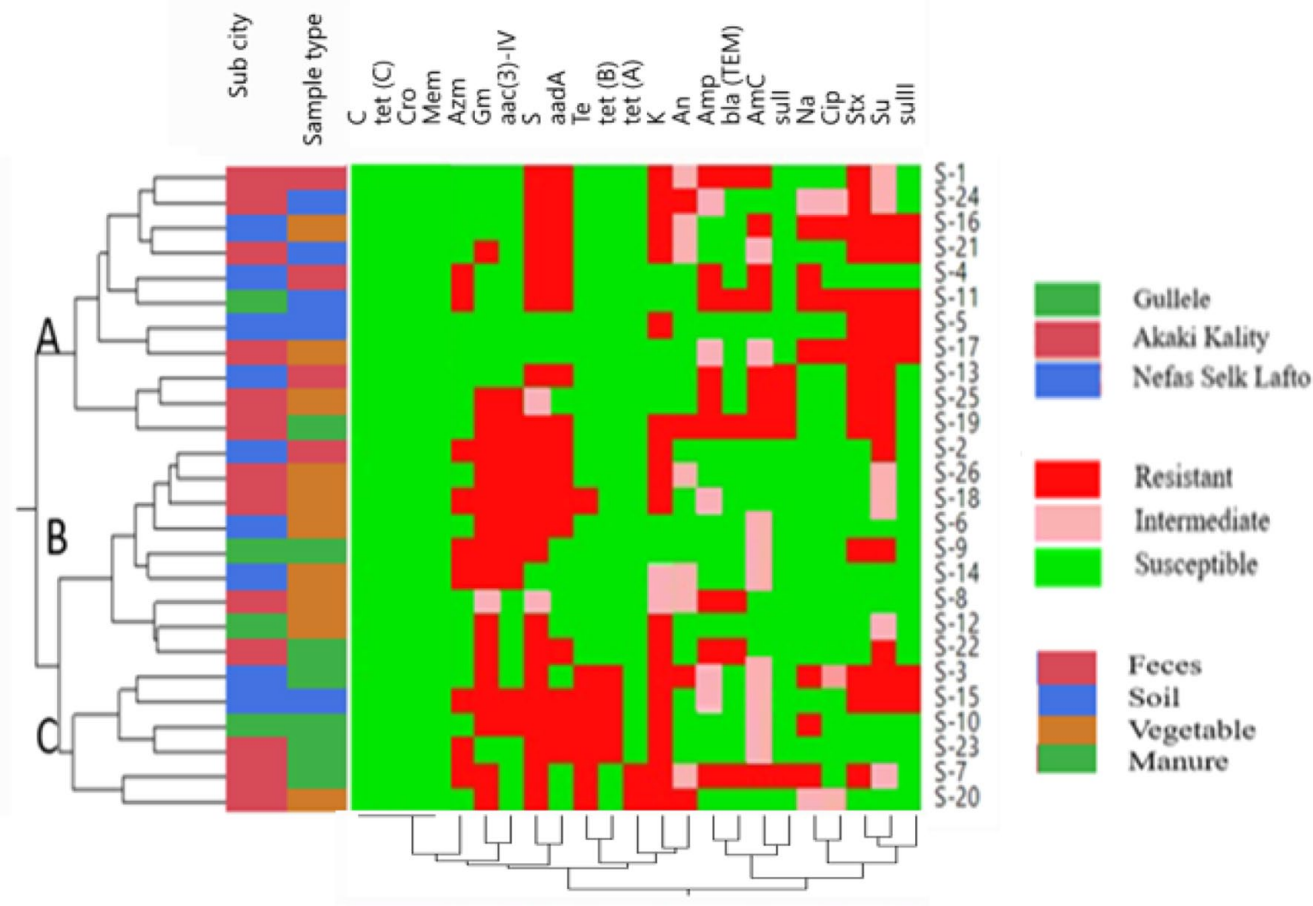
Hierarchal clustering of *Salmonella*. Samples were clustered according to farm, sub-city, sample type, and phenotypic antimicrobial resistance profile. *Amp* ampicillin, *Na* nalidixic acid, *Sxt* sulfamethoxazole + trimethoprim, *Su* sulfisoxazole, *Amc* amoxicillin + clavulanic acid, *S* streptomycin, *K* kanamycin, *Cip* ciprofloxacin, *Te* tetracycline, *Gm* gentamycin, *An* amikacin, *C* chloramphenicol, *Mem* meropenum, *Cro* ceftriaxone, *Azm* azithromycin.

Hierarchal clustering revealed that all the *Salmonella* isolates from the manure and feces samples were resistant to streptomycin. Similarly, all the *Salmonella* isolates from the soil samples were resistant to sulfamethoxazole + trimethoprim. *Salmonella* isolates from fecal samples showed the highest rate of resistance to ampicillin and amoxicillin + clavulanic acid. Among *Salmonella* isolates from manure samples, the highest rate of resistance was recorded for gentamicin and kanamycin. However, based on antimicrobial resistance patterns, isolates with different resistance profiles were shown to be distributed across farms, sub-cities, and specimen types. The antimicrobial susceptibility profiles of *Salmonella* isolates from different sub-cities, and sample types are summarized in Fig. 3.

According to the correlation analysis, antimicrobial agents of the same antimicrobial class were strongly and positively correlated. Additionally, phenotypic resistance is positively and strongly correlated with genotypic resistance. For instance, in the aminoglycoside class of antimicrobials, streptomycin and kanamycin (r = 0.98) were strongly and positively correlated with corresponding resistance genes. Sulfamethoxazole + trimethoprim and sulfisoxazole were strongly and positively correlated (r = 0.9). Moreover, antimicrobials from different classes were correlated with each other. For instance, tetracycline and ampicillin were strongly and positively correlated (r = 0.9). Genotypic resistance to tetracycline was and strongly correlated with *tet (A)* and *tet (B).*

## Discussion

Food safety has come a major public health concern driven by widespread outbreaks of *Salmonella, E. coli* O157, and *Campylobacter*^31^. Fresh vegetables can be contaminated at various stages, from farm to fork^32^. Leafy vegetables are associated with foodborne disease outbreaks, mainly due to *Salmonella* and *E. coli* infections^33^. These outbreaks could be attributed to environmental reservoirs of pathogens and possibly contaminated soil^34^.

Of the vegetable farms included in the current study, 24.6% used animal manure directly without any treatment, which is lower than that reported previously^35^. This result is greater than a report from Minnesota, where only 2% of the farmers applied raw manure^36^. The application of raw manure as fertilizer to vegetables could contribute to vegetable contamination because it is a common source of *E. coli, Salmonella, Campylobacter,* and *Listeria spp.*^37^.

Only 19.8% of the farms were fenced, and the majority were easily accessible by domestic and wild animals in the current study, which is lower than the 60% in Nigeria^38^ and 23% in the US^36^. Fencing vegetable farms is an important agricultural practice because both wild and domestic animals are sources of contamination with foodborne pathogens^39^.

Untreated wastewater is widely used in urban and peri-urban areas of developing countries^40^. In Ethiopia, a large volume of wastewater is released into water bodies that farmers use for irrigation^41^. In this study, rivers were the main source of water for 92.5% of the farms, which is higher than that reported in another study^35^. Moreover, none of the farms protect against the contamination from the water used for irrigation. In previous studies in Nigeria and South Africa, contamination level of water used for irrigation was higher than the WHO standard^42,43^. In addition, studies in Ethiopia showed that dirty water used for irrigation is the main source of contamination^23,44^.

The prevalence of *Salmonella* in this study (2.3%) is lower than that in reports from different countries^35,45,46^ and higher than that in other studies^47,48^. The variability of the prevalence of foodborne pathogens across different geographical areas could be due to seasonal variability and the level of GAP implementation on vegetable farms^35^. In the present study, the prevalence of *Salmonella* varied across the four sub-cities; some of these variabilities could be attributed to their geographic location, the concentration of farms in each location, differences in the number of farms represented, and their agricultural systems^49,50^.

The prevalence of *Salmonella* in the feces of dairy cattle in the current study (5.3%) was relatively higher than reports from previous studies in Ethiopia which ranged from 1.6 to 4.7%^14,51–53^ and lower than the 10.7% prevalence in another study in Ethiopia^54^. These differences might be due to variations in living and housing conditions and the type of feed provided to cattle^55^. The difference in the way feces were collected could also be the reason for the variation. In the current study, fresh feces were collected from the ground, while in some of the previous studies; feces were collected directly from the rectum of the animals.

The prevalence of *Salmonella* in vegetables (2.5%) in the current study was lower than previous report from China (3.4%)^56^. The prevalence of *Salmonella* in lettuce in the current study is higher than the report from a study in Rwanda (1%)^35^. Lettuce is the most consumed leafy vegetable, and *Salmonella* is one of the common pathogens identified in lettuce^57^.

The prevalence of *Salmonella* in soil and manure in the current study was lower than in the US^24,58^. Agricultural animal feeding regimens can affect manure composition and the survival of pathogens in manure^59^. Contaminated manure plays an important role in contaminating agricultural soil, irrigated water, and vegetables, and it enables the microorganisms to survive in soil for several months^37^.

The highest resistance rates were recorded for streptomycin, kanamycin, and gentamicin. Development of resistance to these antimicrobials poses significant public health consequences. High rate of resistance was recorded to ciprofloxacin in the current study. According to WHO classification, fluoroquinolones including ciprofloxacin is recommended to be used only in humans due to its high risk of antimicrobial resistance^60^. Detection of such high level of resistance to this antimicrobial might be due to contamination of the environment by microorganisms from public health facilities. Multidrug-resistant *Salmonella* is one of the leading veterinary and public health problems worldwide^61^. In this study, 61.5% of the *Salmonella* isolates were multidrug-resistant. This finding was lower than that reported in China (92.9%)^47^, and was higher than that reported in Ethiopia and elsewhere (17.9%–58.7%)^24,51,52^. The difference in the use of veterinary antimicrobials may be the reason behind the observed differences.

Sulfonamides, tetracyclines, and fluoroquinolones persist longer in soil and are detected in farm environments^62^. In addition to creating selection pressure on microorganisms in the soil and produce, veterinary antimicrobials could be transferred to the produce, contributing to antimicrobial resistance and possible toxicity in humans consuming these produces^63^. The application of animal manure on vegetable farms is recognized as an important route of transmission for antimicrobial resistance to foodborne pathogens^64^. Contamination of farmlands with antibiotics used for veterinary purposes is a global concern^65^.

The occurrence of a high rate of resistance to gentamicin, kanamycin, and streptomycin in this study was strongly and positively correlated, and the occurrence of  sulfamethoxazole + trimethoprim and sulfisoxazole was strongly and positively correlated. This correlation could be due to the co-selection of resistance to these antimicrobials in *Salmonella* isolates and some of the resistance genetic markers may also be carried on same mobile genetic elements^66^. Studies have shown that there is clonal relatedness among multidrug-resistant *Salmonella* isolates in manure and soil, which highlights the potential role of manure application in the dissemination and persistence of multidrug-resistant *Salmonella*^24^.

## Conclusion

Animal manure is a reservoir for different foodborne pathogens. Our results showed that vegetable farms that applied animal manure as fertilizer were contaminated with *Salmonella*. Moreover, most of these *Salmonella* isolates were multidrug-resistant. Good Agricultural Practice (GAP) was designed to address the source of vegetable contamination, but limited GAP efforts are being made in Addis Ababa to reduce food safety risks. Therefore, there is a need to treat and implement appropriate measures, including composting, before applying animal manure to agricultural farms. In addition, protecting farms from contamination by foodborne pathogens is recommended.

## Data Availability

The authors confirm that all the data are included in the manuscript.
